# Greek Traditional Dance as a Culturally Integrated Intervention: Effects on the Psycho-Emotional Well-Being of Paediatric Nurses During the COVID-19 Pandemic

**DOI:** 10.7759/cureus.102319

**Published:** 2026-01-26

**Authors:** Vasiliki Petraki, Efrosyni Vlachioti, Nikolaos Diggelidis, Antonia Paschali, Vasiliki Matziou

**Affiliations:** 1 Department of Nursing, National and Kapodistrian University of Athens, Athens, GRC; 2 Department of Nursing, "Agia Sophia" Children's Hospital, Athens, GRC; 3 Department of Physical Education and Sport Science, University of Thessaly, Larisa, GRC

**Keywords:** covid-19, greek traditional dance, mental health, paediatric nursing, resilience, well-being

## Abstract

Background: The COVID-19 pandemic has placed an unprecedented psychological and emotional burden on paediatric nurses, who have faced increased workload, increased stress, and burnout. Developing strategies to strengthen the mental resilience and well-being of healthcare professionals has become imperative. Dance, specifically Greek traditional dance (GTD), is a culturally embedded activity with proven benefits on physical, psychological, and social health.

Aim: This study aimed to assess the impact of a GTD training program on the psycho-emotional well-being of paediatric nurses during the COVID-19 pandemic.

Methods: A quasi-experimental study was conducted with a sample of 199 volunteer paediatric nurses, employed in the largest paediatric hospital in Athens, Greece. The participants were divided into an intervention group (Group A) (n=89; 44.7%), who attended 16 training sessions (two sessions per week for two months, lasting 45 minutes each), and a control group (Group B) (n=110; 55.3%), without intervention. Psycho-emotional status was assessed before and after the intervention using the following tools: Positive and Negative Affect Scale (PANAS), Psychological Well-Being Scales-Short Scales (PWBS), Life Satisfaction Scale (SWLS), and Life Orientation Test-Revised (LOT-R). Data analysis was performed with IBM SPSS Statistics for Windows, Version 26.0 (IBM Corp., Armonk, New York, United States), using Cronbach's alpha for reliability, t-test, ANOVA, and non-parametric correlations.

Results: Most participants were women (96% in Group A and 92% in Group B). After completing the program, nurses in the traditional dance group showed significantly greater improvements in positive affect compared to the control group (M±SD=3.89±0.45) and a decrease in negative emotions (M±SD=1.65±0.47) compared to Group B (M±SD=1.94±0.63). At the same time, improvements were recorded in life satisfaction (M±SD=5.30±0.89), optimism (M±SD=3.02±0.72), and relaxation (M±SD=2.48±0.91). The greatest benefits were found in nurses working in wards with paediatric patients positive for COVID-19, who showed an improvement in both positive emotions and a reduction in negative ones, as well as in indicators of friendship and life satisfaction.

Conclusions: Participation in a standardized eight-week GTD program improved the psycho-emotional well-being of paediatric nurses during the COVID-19 pandemic. The results highlight the value of culturally relevant, group-based interventions in promoting the resilience and quality of life of healthcare professionals.

## Introduction

The COVID-19 pandemic has placed unprecedented challenges on healthcare systems worldwide, testing the resilience of nursing staff and placing a significant strain on their psychosocial well-being [1,2]. Paediatric nurses have been facing increased workload, uncertainty, and the unique responsibility of caring for paediatric patients and their families in an unpredictable healthcare environment [3]. Research has documented increased levels of anxiety, depression, emotional exhaustion, and burnout among nurses during the pandemic, highlighting the need for interventions to enhance resilience [4,5].

Although occupational stress and emotional strain are not new phenomena in nursing, the COVID-19 pandemic has exacerbated existing problems, leading to declines in job satisfaction, work performance, and heightened turnover intention [4,6]. Promoting the psycho-emotional well-being of nurses and developing innovative and holistic support strategies are crucial for both the sustainability of the profession and the quality of care provided [7].

Physical activity is widely recognized as an effective means of improving mental health and reducing stress [8,9]. Among forms of exercise, dance stands out as it combines physical activity with artistic expression and social interaction. Dance-based interventions have been associated with improved mood, reduced depressive symptoms, enhanced quality of life, and the promotion of social cohesion in different groups and settings [10,11]. Furthermore, dance offers experiences of collectivity, mutual support, and emotional expression [12].

Greek traditional dance (GTD) occupies a distinctive place in this context. It is a fundamental element of Greek cultural heritage and functions not only as a form of physical activity but also as a vehicle of identity, community, and collective memory [13,14]. Evidence has shown that participation in GTD strengthens social bonds, reduces isolation, and promotes a sense of belonging [13,15]. These cultural and psychosocial elements make GTD an attractive intervention for healthcare professionals, especially in times of crisis.

Despite the above, the literature examining the role of culturally embedded interventions, such as GTD, in supporting the psychosocial well-being of nurses is limited. Most studies focus on general physical activity or mindfulness programs, with few exploring dance as a holistic psychosocial intervention for healthcare staff. In Italy, Dimonte et al. demonstrated that dance movement therapy improved relational and communication skills among nursing students, indicating its potential to enhance core professional competencies [16]. Similarly, Collier and Thompson highlighted both the opportunities and barriers to integrating dance therapy into nursing practice, advocating its use as a supportive tool for staff well-being [17]. In Greece, no studies have been published yet examining the impact of GTD on the psycho-emotional well-being of nursing staff. The existing Greek literature focuses mainly on students or community samples, highlighting benefits in physical condition and social development [14,15]. Consequently, the present study represents the first attempt to implement a culturally integrated dance intervention in paediatric nurses, thus filling a significant research gap in both the national and international literature. From this perspective, the research not only extends the findings of previous studies but also offers new evidence on the role of culturally relevant, non-pharmacological interventions in enhancing the mental resilience of nurses, especially during periods of crisis, such as COVID-19. 

The aim of this study was to assess the impact of a GTD training program on the psycho-emotional well-being of paediatric nurses during the COVID-19 pandemic. Specifically, the study evaluated changes in emotional state, psychological well-being, life satisfaction, and optimism.

## Materials and methods

Study design

The study employed a quasi-experimental design with an intervention group and a control group. Data collection was conducted during the COVID-19 pandemic (2021-2022) at "Agia Sophia" Children's Hospital, the largest paediatric hospital in Athens, Greece. The study was approved by the National and Kapodistrian University of Athens (approval number: 3212) and conducted in accordance with the Declaration of Helsinki.

Sample

A total of 199 paediatric nurses participated. Of these, 89 (44.7%) were included in the intervention group (Group A) and 110 (55.3%) in the control group (Group B). The majority were women (96% in Group A and 92% in Group B). Inclusion criteria included active employment in paediatric departments and provision of informed consent to participate.

GTD intervention 

Group A attended 16 training sessions (two sessions per week for two months, lasting 45 minutes each). The sessions included three parts: warm-up phase (five minutes), GTD training from various regions of Greece (Pontus, Thrace, Macedonia, Epirus, Thessaly, Aegean islands, Peloponnese, Asia Minor), and cool-down/recovery phase (10 minutes). The selected dances were intentionally chosen to be of a low degree of difficulty, with mild and moderate intensity, as they were monitored via perceived exertion (Borg scale 11-13), accompanied by pleasant lyrics and melodious lyrics, ensuring accessibility and enjoyment for all participants.

Measurement tools

Four valid and reliable tools were used to assess the psycho-emotional state: (1) PANAS (Positive and Negative Affect Schedule) for measuring positive and negative emotions, developed by Watson et al. and validated in Greek by Daskalou and Sigkollitou [18,19]; (2) PWBS (Psychological Well-Being Scales) for psychological well-being, developed by Ryff and validated in Greek by Leontopoulou and Triliva [20,21]; (3) SWLS (Satisfaction With Life Scale) for life satisfaction, developed by Diener et al. and validated in Greek by Galanakis et al. [22,23]; and (4) LOT-R (Life Orientation Test-Revised) for optimism developed by Scheier et al. and validated in Greek by Lyrakos et al. [24,25]. Permissions for the use of all instruments were obtained.

Statistical analysis

Data analysis was performed with IBM SPSS Statistics for Windows, Version 26.0 (IBM Corp., Armonk, New York, United States). Paired samples t-test and ANOVA were performed to compare pre- and post-intervention outcomes. Additionally, non-parametric correlations (Spearman's rho) were applied. The statistical significance level was set at p<0.05.

## Results

Most participants were female (96% in Group A and 92% in Group B). The mean age ranged from 35 to 54 years (Group A M±SD=47.02±9.18; Group B M±SD=43.89±11.01), with most nurses working in departments with increased demands during the pandemic (Table 1).

**Table 1:** Demographic characteristics

Characteristic		Group A (n=89)	Group B (n=110)
Mean age (±SD)		47.02 (±9.18)	43.83 (±11.01)
Sex	Female	85 (96%)	101 (92%)
	Male	4 (5%)	9 (8%)
Experience (years, M±SD)		20.80 (±11.85)	17.95 (±11.14)
Experience in the last department (years, M±SD)		11.20 (±9.96)	7.61 (±7.70)
Position	Staff nurse	59 (66%)	85 (77%)
	Head nurse	17 (19%)	11 (10%)
	Coordinator	13 (15%)	14 (13%)
Hospitalization with paediatric patients		57 (64%)	55 (50%)

Following the completion of the GTD training program, the control group showed no statistically significant changes in any outcome measure from baseline to follow-up (all p>0.05).

Positive affect was positively associated with participation in the intervention, while negative affect showed an inverse association, indicating that engagement in the program corresponded with reductions in negative emotional states. Similarly, life satisfaction displayed a positive correlation with the intervention, reflecting enhanced perceptions of overall well-being among participants.

Within the domain of dispositional optimism, positive expectations were positively correlated with intervention participation, whereas negative expectations demonstrated a negative association.

When examining professional roles, staff nurses and head nurses showed positive associations between intervention participation and improved emotional well-being, with each role benefiting in slightly different domains. 

Nurses working in paediatric COVID-19 units exhibited the strongest positive correlations across emotional and social well-being indicators, while those working in non-COVID-19 units showed a more limited association, primarily reflecting improvements in positive emotions.

The main findings are presented in Table 2. 

**Table 2:** Correlations between Group A and Group B post-intervention *p<0.05: statistically significant difference between the intervention group and the control group. **p<0.05: statistically significant difference for the intervention group before and after the intervention.

Variable	Group A (pre) (M±SD)	Group A (post) (M±SD)	Control group (M±SD)
PANAS _PosAffect	3.63±0.57	3.89±0.45*,**	3.62±0.53
PANAS _NegAffect	1.93±0.57	1.65±0.47*,**	1.94±0.63
PWBS _Autonomy	3.73±0.47	3.70±0.53	3.68±0.54
PWBS _Domination	4.06±0.42	4.09±0.45	4.01±0.46
PWBS _Development	4.08±0.36*	4.13±0.47*	3.90±0.49
PWBS _PosRelation	3.82±0.44	3.79±0.44	3.81±0.58
PWBS _Purpose	3.74±0.40*	3.81±0.47*	3.64±0.50
PWBS _Acceptance	3.60±0.45	3.62±0.44	3.58±0.53
SWLS _Satisfaction	5.04±1.00*	5.30±0.89*,**	4.59±1.08
LOT-R _PosExpect	2.89±0.82*	3.02±0.72*,**	2.58±0.79
LOT-R _NegExpect	1.39±0.90*	1.34±0.85*	1.72±0.92

## Discussion

The present study aimed to investigate the impact of a GTD program on the psycho-emotional well-being of paediatric nurses during the COVID-19 pandemic. The results revealed significant improvements in multiple parameters of psychological well-being in the intervention group, supporting the role of dance as a non-pharmacological, culturally integrated intervention.

Our findings are consistent with international studies that have investigated the value of traditional dance forms on psychosomatic health. In Hawaii, participation in a hula program was associated with improved blood pressure control and reduced cardiovascular risk, underscoring the potential of cultural interventions in health promotion [26]. In Argentina, tango has been used as a therapeutic method for people with depression and anxiety, demonstrating improvements in mood and quality of life [27]. Similarly, in Spain, flamenco has been shown to be effective in enhancing emotional balance in older women [28], while in Latin American immigrant populations in the United States, the BAILAMOS program enhanced physical activity and psychological resilience [29]. Placing our results in this broader international context highlights that GTD can function as a corresponding culturally integrated intervention in Greece. The strength of the GTD lies in the fact that it combines physical activity, cultural identity, and social cohesion, elements that may further enhance psychological resilience in crisis conditions.

Recently, published evidence has shown that nurses suffered significant psychological stress during the COVID-19 pandemic, with increased levels of anxiety, depression, burnout, and intention to leave the profession [4,6]. In line with these findings, our study showed that participation in a GTD program reduced negative emotions and increased positive ones, optimism, and life satisfaction. These findings are consistent with research that has shown that participation in dance programs enhances mood and social cohesion [28], reduces levels of anxiety and depression [27], and acts as a protective factor against mental fatigue in healthcare professionals [17]. Therefore, our study adds novel evidence to the field, indicating that GTD can be a feasible, non-pharmacological approach to enhance the psychological resilience of paediatric nurses, both during and beyond crisis periods.

A particularly important finding was that nurses working in wards with COVID-19-positive paediatric patients showed the greatest benefits. This is consistent with the literature, where frontline healthcare workers reported the highest levels of psychological stress [6]. The significant improvement recorded in this subgroup reinforces the view that dance interventions are particularly useful for health professionals experiencing high stress burden.

Furthermore, the differentiation of results according to the job position is notable. Staff nurses showed improvement in multiple parameters (positive emotions, reduction of negative emotions, optimism, life satisfaction), whereas head nurses and coordinators exhibited more specific benefits, such as a sense of friendship and relaxation. This differentiation is linked to the existing literature, which reports that professional responsibilities and roles influence the way nurses experience stress and develop coping strategies [30].

The contribution of the cultural element is crucial. Like tango in Argentina and flamenco in Spain, dance is not simply a form of exercise but an expression of culture, identity, and collective memory. These dimensions enhance a sense of belonging, social cohesion, and psychological empowerment, which amplify the therapeutic value [27,28].

The results of this study offer important practical implications, demonstrating that nurses can benefit from non-pharmacological interventions that utilize elements of local culture. Also, the results emphasize the need to integrate such interventions into organized programs to empower nursing staff, especially in times of crisis, underscoring the importance of long-term strategies that promote mental resilience, so that nurses remain productive and mentally healthy even in conditions of prolonged stress.

Although our study was the first one to show the effectiveness of a GTD training program on the well-being of nurses, it is not without limitations. The quasi-experimental design limits the generalizability of the findings. Self-report questionnaires can introduce risks of response bias, social desirability bias, and expectancy effects. Furthermore, the study was conducted in a single paediatric hospital in Athens, which limits the possibility of drawing conclusions for the entire paediatric nursing population and nurses in general, while the nurses who chose to participate in the dance program may have been more motivated, healthier, or more psychologically resilient at baseline. Nevertheless, a priori power analysis (G*Power v3.1, Heinrich-Heine-Universität Düsseldorf, Düsseldorf, Germany) indicated that a total sample of 180 participants would provide 80% power to detect a medium effect size (f=0.25) at α=0.05.

## Conclusions

Overall, the present study demonstrates that GTD is an effective, culturally adapted intervention that can improve the psycho-emotional well-being of paediatric nurses. Its contribution is not limited to physical exercise but extends to social cohesion and the strengthening of cultural identity. The long-term value of such an intervention lies in its potential to enhance the mental resilience of nurses, better equipping them to face the challenges of both the current pandemic and future health crises.

From a policy and organizational perspective, the results support the inclusion of culturally adapted, non-pharmacological interventions within institutional strategies for mental health promotion among healthcare professionals. Our study is the first attempt to implement GTD in nursing staff in Greece and opens new avenues for future research which should focus on longitudinal designs, larger and more diverse samples, and comparative studies examining GTD alongside other psychosocial interventions. Overall, this study provides an initial framework for the systematic incorporation of culturally grounded interventions into holistic support models for healthcare professionals.
